# Antibacterial, antioxidant and optical properties of edible starch-chitosan composite film containing *Thymus kotschyanus* essential oil

**Published:** 2012

**Authors:** Tooraj Mehdizadeh, Hossein Tajik, Seyed Mehdi Razavi Rohani, Abdol Rassol Oromiehie

**Affiliations:** 1 *Department of Food Hygiene and Quality Control, Faculty of Veterinary Medicine, Urmia University, Urmia, Iran; *; 2 *Department of Plastics, Iran Polymer and Petrochemical Institute, Tehran, Iran.*

**Keywords:** *Essentialoil*, *Thymuskotschyanus*, *Compositefilm*, *Starch*-*chitosan*, *Antibacterial*

## Abstract

Thyme Essential oils (EO) with antimicrobial and antioxidant properties are widely used in pharmaceutical, cosmetic, and perfume industry. It is also used for flavoring and preservation of several foods. Nowadays, packaging research is receiving a considerable attention due to the development of eco-friendly materials made from natural polymers such as starch and chitosan. In this study *Thymus kotschyanus* EO concentrations ranging from 0 to 2.0%, incorporated in starch-chitosan composite (S-CH) film were used. Antimicrobial and antioxidant properties significantly increased with the incorporation of EO (*p* < 0.05). Incorporating EO, increased total color differences (DE), yellowness index (YI) and whiteness index (WI) which were significantly higher than control and its transparency was reduced. Our results pointed out that the incorporation of *Thymus kotschyanus* EO as a natural antibacterial agent has potential for using the developed film as an active packaging.

## Introduction

In the last few years, there has been a growing interest in bio-based polymer packaging products made from raw materials and originating from natural agricultural, marine and livestock raising and renewable sources. Edible films and coatings, prepared from polysaccharides, proteins and lipids have a variety of advantages over synthetic materials, such as biodegradability, edibility, biocompatibility and environmentally friendly.^1^ These packaging materials moreover can serve as a carrier for nutrients, anti-browning agents, flavors and colorants to improve food quality and functionality, and other active ingredients such as antimicrobial and antioxidant compounds for extending product shelf life and reducing the risk of pathogen growth.^2^ These aims achieved with maintaining effective concentrations of active compounds on food surfaces.^3^ This type of packaging that is an innovative concept in food industries is named “Active packaging”.^4^

Starch is a water-soluble polysaccharide with well-known biodegradable and edible film-forming properties. Starch-based packaging materials are widely available in a variety of botanical sources such as corn, wheat, potatoes, yam and tapioca and can be produced at low cost and at large scale from different surplus of harvesting and raw material industrialization.^5^

Chitosan, a biopolymer with unique biodegradability and bioactivity properties, is obtained by partial deacetylation of chitin, Earth's second most widespread amino polysaccharide after cellulose. It is commercially available from byproduct of the seafood industry in large scale because of its abundance in the exoskeleton of shellfish such as shrimp, lobster, crab and other sources.^6^^, ^^7^ Several studies have indicated the antimicrobial and antioxidant characteristics and non-toxicity of chitosan. In addition chitosan possess immense advantages as an edible packaging material owing to its good film-forming properties.^8^ However, wide application of starch film is limited by its water solubility and brittleness. Therefore, chitosan films have relatively poor water vapor barrier characteristics.^9^ One of the effective strategies to overcome the poor mechanical properties of these films, while preserving the biodegradability of the materials, is composite films that can be formulated to combine the advantages of each component.^10^^, ^^11^

There have been consumer demands for more natural preservatives, mainly because of safety concerns, in that the residual chemicals might be hazardous. In this regard incorporation of natural preservatives such as plant extracts and essential oils with antimicrobial and antioxidant properties, into bio-based packaging materials, provide an innovative means that improve safety and shelf life of food.^4^^, ^^12^

The aromatic and medicinal properties of the genus *Thymus* have made it one of the most popular plants throughout the entire world. Thymus essential oils and extracts with antimicrobial and antioxidant properties are widely used in pharmaceutical, cosmetic, herbal tea, flavoring agents and perfume industry, also for flavouring and preservation of several food.^13^ Because of the effect of direct addition of essential oils to food on sensory characteristics of added food, incorporation of essential oils to edible films may have supplementary applications in food packaging.^14^^,^^15^ There are some studies dealing with the antimicrobial properties of films based on starch or chitosan incorporated with various essential oils with good results.^16^^-^^22^ However, information about either the incorporation of *Thymus kotschyanus* EO in starch – chitosan (S-CH) composite films has not been found. The objective of this study was to prepare composite films from S-CH, incorporated with *Thymus kotschyanus* essential oil and to evaluate their antibacterial, antioxidant and optical properties.

## Materials and Methods

**Plant material and Gas chromatography mass spectrometry (GC–MS) analysis. **The dried Leaves and aerial parts of *Thymus kotschyanus* was purchased from local grocery store and authenticated at the department of horticulture, faculty of agriculture, Urmia University, Urmia, Iran. Essential oil was obtained by hydro-distillation for 3 h using a Clevenger-type collector. The obtained EO was hydrated with sodium sulfate then filtered through 0.22 µm (Millipore™, Bedford MA, USA) and stored in airtight glass vials covered with aluminum foil at 4 ˚C. The constituents of EO were identifying by GC-MS (Thermo-UFM, Milan, Italy). 

**Preparation of films. **Chitosan-based film was prepared by dissolving medium molecular weight, crab shell chitosan (~400kDa, 75–85% deacetylated) (Fluka, Sigma-Aldrich, St. Louis MO, USA) in an aqueous solution (1% v/v) of glacial acetic acid (Merck, Darmstadt, Germany) to a concentration of 2% (w/v) while stirring on a magnetic stirrer-hot plate. The solution was stirred with low heat (at 50 ˚C) which typically required 3 h stirring. The resultant chitosan solution was filtered through a Whatman No. 3 filter paper and followed by vacuum filtration to eliminate insolubles and remove any undissolved particles. Starch solutions with concentrations of 3.5 % (w/v) were prepared by dispersing 27% amylose corn starch (Sigma-Aldrich Chemie GmbH, Steinheim, Germany) in distilled water and heating the mixtures on hotplates 95 ˚C during 30 min with stirring until it gelatinized, and then cooling to 40 ˚C.^23^

Starch-Chitosan composite films were prepared by mixing 100 mL of 2% chitosan solution with 100 mL of 3.5% starch solutions. Glycerol (Sigma Chemical Co., St. Louis, MO, USA) was added as 30% (w/w) of the total solid weight in solution. Tween 80 at level of 0.2% (v/v) of EO was added in film forming solutions to assist essential oil dissolution, and then EO was added to the S- CH solution to reach a final concentration of 0%, 0.5%, 1% and 2% (w/w). The solution was homogenized (IKA T25 basic, Staufen, Germany) at 8000 rpm for 3 min to obtain an emulsion. The mixtures were cast on to flat, level polytetrafluoroethylene casting plate. After drying the films at room temperature for at least 72 h, they were peeled from the plates. Dried films were conditioned at 50% RH and 25 ˚C for 48 h prior to testing.

**Determination of antibacterial effects of films.** For the antibacterial activity test, *Staphylococcus aureus* (ATCC 25923)*, Salmonella typhimurium *(ATCC 1709)*, Escherichia coli *O157:H7 (ATCC 25922) and* Listeria monocytogenes* (ATCC 1915) from culture collection of the Department of Food Hygiene and Quality Control, Faculty of Veterinary Medicine, Urmia University, Urmia, Iran were used. The bacterial cultures were grown on the nutrient agar slant and kept at 4 ˚C. Monthly subculture was carried out to maintain bacterial viability. In the preparation of seeding culture, a loopful of bacteria from agar slant was taken and inoculated into 50 mL of tryptic soy broth (Merck, Darmstadt, Germany) in 125 mL flask. The flask was then incubated at 125 rpm in an incubator at 37 ˚C for 24 h. A dilution series was taken to meet required bacterial population for seeding by using sterile distilled water. The agar diffusion method was used for determining the antibacterial effects of films on bacterial strains. Disks (12 mm diameter) cut from the films were placed on Mueller Hinton agar (Merck) plates, previously surface spread with 0.1 mL of inoculums containing approximately 10^5^–10^6^ CFU mL^-1^ of tested bacteria. The plates were then incubated at 37 ˚C for 24 h. The diameter of the zone of inhibition was measured with a caliper to the nearest 0.01 mm. The whole zone area was calculated then subtracted from the film disc area and this difference in area was reported as the ‘‘zone of inhibition”. The contact area was also examined visually to evaluate growth inhibition underneath the film disk contact.^24^

**Determination of antioxidant activity. **The antioxidant activity of the film samples was evaluated using DPPH (2, 2-diphenyl-1-picrylhydrazyl) free radical scavenging assay. Briefly, 3 mL of film extract solution were mixed with 1mL of 1 mM methanolic solution of DPPH (Merck, Darmstadt, Germany). The mixture was vortexed and incubated in the dark at ambient temperature for 30 min. When the DPPH solution was mixed with the sample mixture acting as a hydrogen atom donor, a stable non radical form of DPPH is obtained with simultaneous change of the violet color to pale yellow. The absorbance was then measured at 517 nm. The percentage of DPPH free radical quenching activity was determined using the following equation:

$\mathrm{DPPH scavenging effect \%}=\frac{\mathrm{AbsDPPH}–\mathrm{AbsExtract}}{\mathrm{AbsDPPH}}\times 100$

Where Abs DPPH is the absorbance value at 517 nm of the methanolic solution of DPPH and Abs extract is the absorbance value at 517 nm for the sample extracts. Each sample was assayed at least five times.^25^

**Total phenolic assay. **For this purpose, 25 mg of each film sample were dissolved in 3 mL of distilled water. Phenolic compound content in each film extract was determined according to the Folin-Ciocalteu procedure as described by Singleton, Orthofer and Lamuela-Raventos with slight modifications by Siripatrawan and Harte.^25^^, ^26 Briefly, 0.1 mL of film extract solution were mixed with 7mL distilled water and 0.5 mL of Foline Ciocalteu reagent (Merck Company, Darmstadt, Germany). The mixture was incubated for 8 min at room temperature before addition of 1.5 mL of sodium carbonate solution and 0.9 mL of distilled water. The mixture was stored in a dark chamber at room temperature for 2h. The absorbance of the mixture was then measured at 765 nm using a spectrophotometer gallic acid solutions (Sigma–Aldrich, USA) in the specific concentration range were used to construct a calibration curve. The concentration of total phenolic compounds in the samples is expressed as gallic acid equivalents (GAE),which reflect the phenolic content as the amount of gallic acid in mg per gram dry weight of the sample, calculated by using an equation that was obtained from the standard graph (R2 = 0.991), is given as:

*Abs*_765_ *= 0.912 mg gallic acid + 0.041*

**Film solubility in water. **A modified method from Jutaporn *et al.* and Rhim *et al*. was used to measure film solubility. Film portions measuring 1×3 cm^2^ were cut and were dried at 110 ˚C in a vacuum oven for 24 h and then weighed to the nearest 0.0001 g for the initial dry weight. Then films were placed in glass beaker with 50 mL of distilled water and shaken gently at 25 ˚C for 24 h. The solution was then filtered through Whatman No. 1 filter paper to recover the remaining undissolved film. The remaining pieces of film after immersion were dried at 110 ˚C to constant weight (Final dry weight). Tests for each type of film were carried out in three replicates.^27^^,^^28^ Solubility in water (%) was calculated by using the following equation:

$\mathrm{Solubility in water}\left(\%\right)=\frac{\mathrm{Initial dry weight}–\mathrm{Final dry weight}}{\mathrm{Initial dry weight}}\times 100$

**Surface color and opacity measurements. **Film color was determined by a colorimeter (Minolta Chromameter cr-400, minolta Co., Ltd., Osaka, Japan). Hunter color scale was used, lightness (*L*) and chromaticity parameters *a* (red–green) and *b* (yellow–blue) were measured. Measurements were performed by placing the film sample over the standard white plate (*L*=91.35, *a*=0.31 and *b*= ˗1.21). Total color difference (ΔE), yellowness index (YI), and whiteness index (WI) were calculated as Bolin and Huxsoll: ^25^

*∆* *E = [(L*_standard_ *-L*_sample_ *)* ^2^ *+(a*_standard_ *-a*_sample_ *)* ^2^ *+(b*_standard_ *-b*_sample_ *)* ^2^ *]* ^0.5^

*YI = 142.86b × L* ^-1^

*WI = 100 – [(100 – L)* ^2^ *+ a* ^2^ *+b* ^2^ *]* ^0.5^

Transparency was determined according to the method of Siripatrawan and Harte by measuring the film absorbance at 600 nm using a UV spectrophotometer.^25^ The films were cut in to a rectangle piece and directly placed in a spectrophotometer test cell. An empty test cell was used as the reference. The transparency of the films was calculated as follows:

*Transparency = log ( T600) x* ^-1^

Where *T*600 is the transmittance at 600 nm and x is the film thickness (mm). According to this equation; the high values of *T* indicate lower transparency and higher degree of opacity.

**Statistical analysis.** The statistical analysis of the data was performed through an analysis of variance (ANOVA) using IBM SPSS Statistics Software (Version 20.0, IBM SPSS Inc, Armonk, NY, USA). Duncan's multiple range test was used to detect differences among mean values of films. The *p*-value of the test was ≤ 0.05.

## Results

**Identification of volatile components from essential oil. **Results of GC–MS analytical data of compounds in *Thymus kotschyanus* EO showed that major constituents were Thymol (GC peak area%, 26.61%), Carvacrol (12.60%), Cis-Geraniol (5.59%), Caryophyllene (5.58%), Germacrene- D (5.03%), Camphor (4.79%), α-terpineol (4.78%), Terpinen-4-ol (4.70%), Eucalyptol (1, 8-Cineole or Limonene) (4.66%), Terpineol, cis-beta-(3.80%), p-cymene (3.43%), γ-terpinene (3.33%).

**Antimicrobial activity of edible S-CH composite films. **The growth inhibition zones were measured using agar disc diffusion assay. Effects of *Thymus kotschyanus* EO addition on antimicrobial properties of S–CH composite based films are shown in Table 1. When antimicrobial agents are incorporated into films, these materials diffuse through agar gel and result in clear zone around the film cuts. *Thymus kotschyanus* EO exhibited different inhibition levels against *L. monocytogenes*, *E. coli O157:H7*, *S. aureus* and *S. typhimurium* as shown in Table 1. In this study, the inhibition zone was increased with increasing concentration of EO, but this was not significant for all concentration in four tested microorganism (*p* ≤ 0.05). A Chitosan-Starch composite film without EO was not effective against *S. typhimurium* and clear zone of inhibition was not observed.

**Fig. 1:**
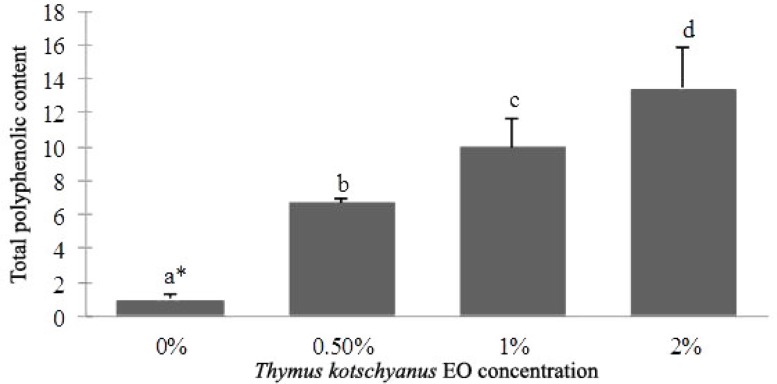
Total polyphenolic content (mg gallic acid) in 1 g of S-CH composite film incorporated with Thymus *kotschyanus* EO. Values are given as mean ± SD. Different letters indicate significantly different (*p* < 0.05) when analyzed by Duncan’s New Multiple Range Test

**Total phenolic content and antioxidant activity. **Foline Ciocalteu phenol reagent is used to find a crude estimate of the amount of phenolic groups present in S-CH composite film. The results showed that total phenolic content in the S-CH films significantly was increased (*p* ≤ 0.05) with increasing EO concentration (Fig. 1).

**Fig. 2:**
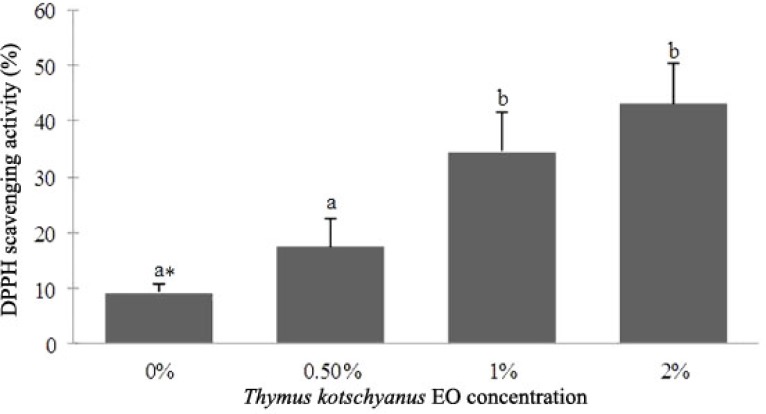
DPPH scavenging of S-CH composite film incorporated with Thymus *kotschyanus* EO. Values are given as mean ± SD. Different letters indicate significantly different (*p* < 0.05) when analyzed by Duncan’s New Multiple Range Test

DPPH scavenging assay was used to indicate antioxidant activity of the film. This assay was based on the ability of DPPH, a stable free radical, to be quenched and thereby decolorize in the presence of antioxidants resulting in a reduction in absorbance values.^25^ The results showed that DPPH scavenging activity of the S-CH films significantly was increased (*p* ≤ 0.05) with increasing EO concentration as shown in Fig. 2.

**Table 1:** Antibacterial activity of edible S-CH composite films incorporated *Thymus kotschyanus* EO against different bacteria

Bacteria	Essential oil concentration (%) in film solution	Inhibitory zone (mm^2^)	Contact area
*Listeria* *monocytogenes*	0	* ^a^ 13.24±35.02	-
	0.5	^a^ 10.20±100.52	-
	1	^b^ 33.11±187.31	-
	2	^c^ 43.25±285.44	-
*Staphylococcus aureus*	0	^a^ 6.12 ± 13.22	-
	0.5	1.63^ a^ ± 19.83	-
	1	6.65^ a^ ±32.90	-
	2	26.32^ b^ ±111.84	-
*Escherichia coli O157:H7*	0	^a^ 3.39±11.43	-
	0.5	^a^ 2.01±24.31	-
	1	^b^ 9.95±44.21	-
	2	^c^ 12.05±163.20	-
	0	^a^ 0.00±0.00	+
*Salmonella typhimurium*	0.5	^a^ 0.91±12.27	-
	1	^a^ 11.23±21.46	-
	2	^b^ 17.07±66.12	-

Contact area is the part of agar on Petri dish directly underneath film pieces.

* indicates in each column with different superscript letters are significantly different (*p* < 0.05).

**Film solubility in water.** The water solubility of the S-CH composite films as a function of EO content is shown in Table 2. Addition of EO, in all concentrations, increased water solubility of films. The percentage of water solubility was 12.54 % for the samples without EO, which was increased to 23.29 for the films containing 2% EO. However, significant (*p* ≤ 0.05) increase in solubility was observed at the high levels of EO. 

**Surface color and opacity. **The effects of EO concentration on *L*, *a* and *b* Hunter Lab color values, total color difference (ΔE), yellowness index (YI), whiteness index (WI) and opacity of films are shown in Table 2. Adding EO into chitosan films significantly affected (*p* ≤ 0.05) *L* (lightness/darkness), *a* (redness/greenness) and *b* (yellowness/blueness) values of the film surface. Films without EO were lighter (higher *L* value). *L* values of the films was decreased from 87.62 to, 80.25 but *a *was decreased from -1.23 to -1.95 (indicator of the tendency towards redness) and *b* values was increased from 11.38 to 17.49 (indicator of the tendency towards yellowness), as the EO concentrations were increased from 0 to 2%.

**Table 2:** Hunter color values (*L*, *a* and *b*), opacity (T), yellowness index (YI), whiteness index (WI), total color difference (ΔE), and Solubility in water of S-CH films as a function of *Thymus kotschyanus* EO concentration

EO (%)	*L*	*a*	*b*	T	WI	YI	ΔE	Solubility in water (%)
0	87.62^ d^*	-1.23^ a^	11.38^ a^	1.03^ a^	83.13^ d^	^a^ 18.55	^a^ 13.13	12.54^ a^
0.5	85.46^ c^	-1.43^ b^	12.01^ b^	2.49^ b^	80.01^ c^	20.79^ b^	^b^ 14.56	16.54^ b^
1	85.46^ c^	-1.84^ c^	13.84^ c^	3.86^ c^	77.15^ b^	^c^ 24.13	^c^ 18.06	20.23^ c^
2	80.25^ a^	-1.95^ d^	17.49^ d^	4.33^ d^	73.58^ a^	31.13^ d^	^d^ 21.84	23.29^ c^

* indicates in each column with different superscript letters are significantly different (*p* < 0.05).

## Discussion

Results of GC–MS analytical data of compounds in *Thymus kotschyanus* EO showed that EO is rich in monoterpene phenols, especially thymol and carvacrol that have antibacterial and antioxidant properties. The results showed that *L. monocytogenes* was the most sensitive bacteria against *Thymus kotschyanus* EO incorporated films, followed by *E. coli *O157:H7, *S. aureus* and *S. typhimurium*. As the concentration of EO increased, the inhibition zone was increased significantly (*p* ≤ 0.05) but this was not significant in 0.5 and 1% concentrations containing film for *S. aureus* and *S. typhimurium*.

The inhibitory effects of essential oils on the types of bacteria such as gram-positive or gram-negative bacteria are still in controversies. Emiroğlu *et al*. determined anti-bacterial activity of soy protein edible films incorporated with oregano and thyme essential oils and showed that while *E. coli*, *E. coli O157:H7* and *S. aureus* were significantly inhibited by antimicrobial films, *L. plantarum* and *P. aeruginosa* appeared to be the more resistant bacteria.^29^ Solomakos *et al.* distinguished that 0.60% thyme essential oil had an inhibitory effect against *E. coli O157:H7* when applied directly in the minced meat during refrigerated storage at 10 ˚C.^30^ Seydim and Sarikus evaluated antimicrobial activity of whey protein isolate based edible films incorporated with essential oils and reported more inhibitory effects of whey protein isolate-based edible film containing 2% oregano oil against *L. monocytogenes* than *E. coli O157:H7*.^24^ Oussallah *et al.* showed that 1% oregano essential oil addition into the milk protein based edible films inhibited *E. coli O157:H7*.^31^ In another study carvacrol containing tomato based edible films inactivated the *E. coli O157:H7*, with the inactivation related to carvacrol levels in the films.^32^ Thymol and carvacrol are thought to be the major active compounds present in thyme and oregano EO and reported to have inhibitory effects against microorganisms through breakdown of the outer membrane of micro-organism and lead to an excessive leakage of essential elements, and cause bacterial death.^33^ The results showed that the *Thymus kotschyanus* EO incorporated in S-CH films exhibited significant inhibitory effects against common foodborne pathogenic bacteria such as *L. monocytogenes*, *E. coli *O157:H7,* S. aureus* and *Salmonella enteritidis.*

Folin-Ciocalteau (FC) colorimetry is based on a chemical reduction of the reagent, a mixture of tungsten and molybdenum oxides. Phenolic compounds undergo a complex redox reaction with phosphotungstic and phosphomolybdic acids present in the Foline Ciocalteu reactant.^34^ On the basis of FC results, total phenolic content in the S-CH films significantly was increased (*p* ≤ 0.05) with increasing EO concentration (Fig. 1). DPPH scavenging assay was used to indicate antioxidant activity of the film. As the concentration of EO increased, DPPH scavenging activity of the films increased significantly (*p* ≤ 0.05) but this was not significant between 0.5 - 1% and 1 - 2% concentrations. In the films containing 2% EO, the antioxidant activity was increased 4.5 folds more than the control samples. In a study by Amiri, *Thymus kotschyanus* EO showed 117 μg GEs per mg of extract Phenolic content and 278 μg mL^-1^ DPPH IC50 antioxidant activity.^35^ In another study chitosan film incorporated with 1% and 2% *Zataria multiflora Boiss* EO exhibited 33.98% and 37.77% DPPH scavenging activity, respectively, and in this manner 5.6 and 11.2 mg gallic acid per gram film Phenolic content.^36^

The chitosan films with no EO showed some scavenging activity on DPPH (9.10%). This is associated with the fact that free radicals can react with the residual free amino (NH2) groups of chitosan to form stable macromolecule radicals, and the NH2 groups can form ammonium (NH_3_^+^) groups by absorbing a hydrogen ion from the solution.^37^ However, results of this study showed that incorporation of GTE in to chitosan films improved enhanced polyphenolic content and antioxidant activity of the films.

In both edible and inedible films, color is an important factor in terms of consumer acceptance. The addition of *Thymus kotschyanus* EO affected the color and transparency of S-CH edible films. Edible S-CH films without EO appeared clear and transparent and S–CH composite films incorporated with EO showed significantly higher ΔE*, b* value (yellowish) and lower *L* value (darker) than control film (*p* ≤ 0.05). In one study, Chitosan-based films containing cinnamon essential oil was investigated by Ojagh *et al*. and similar results were reported.^19^ Pranato *et al*. showed that addition of garlic EO affected the appearance of edible film in both color and transparency. When garlic oil at 0.30% or higher concentration was incorporated, the color tended to yellowish as indicated by the increase of b value. L values were decreased and color of the edible film tended to darken.^14^

Addition of EO, in all concentrations, increased the water solubility of S–CH composite films. Although a higher solubility of edible film is required during cooking food products coated with edible film, a low solubility is required during storage.^38^ Laohakunjit and Noomhorn showed that inclusion 0.40 % lemongrass EO in starch film increased the water solubility of films. This was described by interference of EO with arrangement of polymer chains and hydrogen binding and in this manner less interaction between the starch molecules. Furthermore leaching of amylase from starch component in the film can increase percent of water solubility.^38^ These findings is in contrary with Ojagh *et al*. study that demonstrated, incorporation of CEO into the chitosan film formulation at level of 1.5% and 2% (v/v) led to 41.00% and 55.00% reduction in solubility in water, respectively.^19^

Starch and Chitosan as natural polymers have great potential for usage in bio-based packaging materials. The results showed that incorporation of *Thymus kotschyanus* EO improved the antibacterial and antioxidant properties of S-CH composite film. *Thymus kotschyanus* EO had significant inhibitory effects against four common foodborne pathogenic bacteria used in this study. The color of edible films was darker and more yellowish as the *Thymus kotschyanus* EO increased. 

In conclusion, an antibacterial and antioxidant S-CH composite film incorporated with *Thymus kotschyanus* EO is promising and has good potential to enhance the safety of foods and food products. Future research could be conducted to evaluate the sensory aspects of using these natural essential oil compounds in edible films and coatings, as well as to characterize their stability and other physico-mechanical properties. Moreover, the anti-microbial effect of CEO enriched films should be determined on an entire model food.
